# A chromosome-scale genome assembly and karyotype of the ctenophore *Hormiphora californensis*

**DOI:** 10.1093/g3journal/jkab302

**Published:** 2021-10-16

**Authors:** Darrin T Schultz, Warren R Francis, Jakob D McBroome, Lynne M Christianson, Steven H D Haddock, Richard E Green

**Affiliations:** 1 Department of Biomolecular Engineering and Bioinformatics, University of California Santa Cruz, Santa Cruz, CA 95064, USA; 2 Monterey Bay Aquarium Research Institute, Moss Landing, CA 95039, USA; 3 Department of Biology, University of Southern Denmark, Odense 5230, Denmark; 4 Department of Ecology and Evolutionary Biology, University of California Santa Cruz, Santa Cruz, CA 95064, USA

**Keywords:** ctenophore, Ctenophora, genomics, comb jelly, chromosome-scale, heterozygosity, Iso-Seq, Hi-C, PacBio, inversion

## Abstract

Here, we present a karyotype, a chromosome-scale genome assembly, and a genome annotation from the ctenophore *Hormiphora californensis* (Ctenophora: Cydippida: Pleurobrachiidae). The assembly spans 110 Mb in 44 scaffolds and 99.47% of the bases are contained in 13 scaffolds. Chromosome micrographs and Hi-C heatmaps support a karyotype of 13 diploid chromosomes. Hi-C data reveal three large heterozygous inversions on chromosome 1, and one heterozygous inversion shares the same gene order found in the genome of the ctenophore *Pleurobrachia bachei*. We find evidence that *H. californensis and P. bachei* share thirteen homologous chromosomes, and the same karyotype of 1*n* = 13. The manually curated PacBio Iso-Seq-based genome annotation reveals complex gene structures, including nested genes and trans-spliced leader sequences. This chromosome-scale assembly is a useful resource for ctenophore biology and will aid future studies of metazoan evolution and phylogenetics.

## Introduction

Ctenophore genome assemblies have been key to understanding the early evolution of animals. The draft genomes of the ctenophores *Mnemiopsis leidyi and Pleurobrachia bachei* showed that many important animal developmental and neuron-specific genes did not evolve until the common ancestor of the bilateria (Ryan *et al.* 2013; Moroz *et al.* 2014). Years after publication, these two ctenophore genomes remain crucial for studying the evolution of gene families and developmental pathways in the ancestor to all animals (Sebé-Pedrós *et al.* 2018; Fernández and Gabaldón 2020; Tikhonenkov *et al.* 2020; Wang *et al.* 2020), and for studying the evolution of genome regulation within animals (Gaiti *et al.* 2017; Bråte *et al.* 2018).

It remains controversial whether ctenophores or sponges are sister to the rest of animals (Simion *et al.* 2017; Shen *et al.* 2017; Whelan *et al.* 2017; Laumer *et al.* 2019). Therefore, it is unclear on what ancestral evolutionary branch some metazoan characters evolved, such as neurons and the mesoderm. One method that could possibly resolve the phylogenetic position of ctenophores and sponges is comparing whole-chromosomes (Sacerdot *et al.* 2018). However, the *M.* *leidyi* and *P.* *bachei* assemblies are not chromosome-scale. Furthermore, karyotypes are not known for *M. leidyi*, *P. bachei*, or any other ctenophore.

In contrast to the hundreds of published chromosome-scale genome assemblies from vertebrates and other bilterians, there are currently only three from nonbilaterian animals: the freshwater sponge *Ephydatia* (Kenny *et al.* 2020), the cnidarian *Rhopilema* (Li *et al.* 2020; Nong *et al.* 2020), and the cnidarian *Nematostella* (Zimmermann *et al.* 2020). The disparity in the number of bilaterian versus nonbilaterian chromosome-scale assemblies can be partly explained by the difficulties of isolating nucleic acids from nonbilaterians (Dawson *et al.* 1998; Simister *et al.* 2011). Also, nonbilaterians tend to have highly heterozygous genomes (Leffler *et al.* 2012), which complicates standard approaches to genome assembly (Kajitani *et al.* 2014). The assemblies from *Ephydatia*, *Rhopilema*, and *Nematostella* were possible only due to recent advances in long-read sequencing and the advent of Hi-C data for whole-genome scaffolding (Burton *et al.* 2013; Rice and Green 2019).

*Hormiphora californensis* is a globular 2 cm ctenophore abundant in the temperate Pacific Ocean with several attractive features for experimental work (Matthews and Vosshall 2020). This species is readily cultured in aquaria (Patry *et al.* 2019), has a life cycle as short as 2 weeks, produces hundreds to thousands of eggs per spawning event, and has easily observed embryonic development (Freeman 1977). In addition, there are established CRISPR-Cas9 genome-editing methods for other ctenophore species that may be adaptable to *Hormiphora* (Presnell and Browne 2021). The genus *Hormiphora* is in the same family, the Pleurobrachiidae, as the ctenophore *P.* *bachei*. Given these useful traits, and the availability of the *P. bachei* genome, we selected *H. californensis* for chromosome-scale genome assembly.

Here, we report a karyotype, chromosome-scale genome assembly, and a manually curated genome annotation of *H. californensis* individual Hc1. Using Hi-C data from Hc1 and Hc3, we present evidence for three heterozygous inversions that span 73% of one *Hormiphora* chromosome. We find that there are several inversion breakpoints in common between *Hormiphora and Pleurobrachia*. We estimate the indel and SNP heterozygosity of *H. californensis*. We use Iso-Seq based annotation to resolve hundreds of complex nested intronic (NI) genes, and find that trans-spliced leaders are common in ctenophore mRNAs. The *H. californensis* genome assembly, annotation, and sequencing data will be a valuable resource for comparative genomics and evolutionary studies.

## Materials and methods

### Sample collection

We sampled two *H. californensis* individuals, Hc1 and Hc2, wild-caught from the Monterey Bay (Table 1). We also sampled a third individual, called Hc3, from the seventh generation of a lab-reared culture at the Monterey Bay Aquarium. The aquarium culture’s provenance was a single broadcast spawning event from ten individuals wild-caught in the Monterey Bay. Hc1 and Hc2 samples were collected with the *ROV Ventana* aboard the Monterey Bay Aquarium Research Institute’s *R/V Rachel Carson*, and from a Tucker trawl aboard MBARI’s *R/V Western Flyer*. Samples were flash frozen in liquid nitrogen after allowing the gut to clear. Samples were collected under the State of California Department of Fish and Wildlife collecting permit SC-4029. Additional details are included in Table 1 and Supplementary Figure S1. An individual collected by Tucker trawl, Hc1, was selected as the sole source for DNA and RNA sequencing for the genome assembly and annotation.

**Table 1:** *Hormiphora californensis* sample collection details

Biosample accession	Name	Depth	Collec. temp.	Collection method	Collec. date	Latitude, longitude DMS	mtDNA accession
SAMN12924379	Hc1	0-520m	6°C–13°C	Tucker Trawl	2016-12-13	36° 41' 42'' N, 122° 5' 22'' W	MN544300
SAMN12924380	Hc2	230m	8.9°C	ROV Ventana	2013-11-11	36° 41' 48'' N, 122° 2' 36'' W	MN544301
SAMN16124402	Hc3	0m	12°C	F7 from Monterey Bay Aquarium	2020-06-05	36° 41' 48'' N, 122° 2' 36'' W	NA

### Karyotyping

We prepared *H. californensis* chromosomes from embryos to produce a karyotype. To collect embryos we placed Tucker trawl-collected *H. californensis* individuals in 200 mL of 12°C filtered seawater, adapted the animals to darkness for 4 h, then induced spawning with light (Patry *et al.* 2019). The embryos were concentrated into 10 mL of seawater using a 40 µm mesh Fisherbrand Sterile Cell Strainer, then were incubated at 12°C for 6 h to allow development to approximately the 64-cell stage. The embryos were fixed using a protocol for chromosome spread preparation for *Nematostella vectensis* (Guo *et al.* 2018). The slides of chromosome preparations were stained using DAPI, mounted with Fluoromount-G, then stored at 4°C until imaging. Micrographs of chromosome spreads were collected with a 100x objective and 1.5x diopter on a Leica DM5500 B microscope with a DAPI excitation light and filter at the UC Santa Cruz Life Sciences Microscopy Center.

### Data preparation

In total, we constructed 13 Illumina and PacBio DNA and RNA sequencing libraries. Eleven of these libraries were from the individual used for genome assembly and annotation, Hc1. The remaining two libraries were one Illumina WGS of individual Hc2, and a Hi-C library of individual Hc3. The preparation protocols used for the Chicago (Putnam *et al*. 2016) and Hi-C (Adams *et al*. 2020) libraries were from published methods. Briefly, from Hc1 we collected 247x coverage of PacBio WGS CLR reads, 573x coverage of Illumina WGS reads, 1956x coverage of Chicago and Hi-C reads, 28 Gbp of Illumina RNA-seq reads, and 2.5 million Iso-Seq transcripts. The mean read length for both the PacBio Sequel I CLR and the PacBio Sequel II Iso-Seq data was 2.7 kb (Supplementary Figure S2). The Iso-Seq read length distribution roughly matched the size distribution of the input RNA (Supplementary Figure S3). Sequencing was performed at the University of California Davis (UCD) DNA Technologies Core, Fulgent Genetics, MedGenome Inc., or at the University of Utah. The raw data are available on NCBI under BioProject PRJNA576068. Details for each library are available in Table 2. Reads were trimmed and prepared for genome assembly and genome annotation. For details, see the Supplementary section *Sequencing data preparation*.

**Table 2:** A summary of the *H. californensis* SRAs sequenced for this study

Individual	SRR	Data type	Total GB	Number of reads (or pairs)—Millions	Physical coverage
Hc1	SRR10237148, SRR10237149, SRR10237137	PacBio WGS CLR	27.4	9.7	247.7

	SRR10237134	10X Chromium	22.3	74.3	201.4

	SRR10237129, SRR10237130, SRR10237131, SRR10237132, SRR10237133	Illumina WGS—NEBNext UII	36.1	120.2	325.8

	SRR10237128	Chicago—DpnII	10.7	35.6	96.6

	SRR10237146, SRR10237147	Chicago—MluCI	20.9	69.8	189.2

	SRR10237145, SRR13784183	HiC—DpnII—rep 1	50.0	166.5	451.3
	
	SRR10237144, SRR13784182	HiC—DpnII—rep 2	68.1	226.8	614.8
	
	SRR10237139, SRR13784181	HiC—MluCI—rep 1	38.1	127.1	344.5
	
	SRR10237138, SRR13784180	HiC—MluCI—rep 2	28.8	95.9	260.0
	
	SRR10237136	TruSeq RNA Library Prep Kit v2 (stranded)	28.8	96.0	NA
	
	SRR10403849, SRR10403581	Iso-Seq Express	6.0	2.5	NA

Hc2	SRR10237135	Illumina TruSeq Nano DNA	12.8	64.0	115.7

Hc3	SRR12632403, SRR13784179	HiC—DpnII	70.2	233.9	633.8

Each row is a single library. For individual Hc1, the total physical coverage of DNA WGS reads is 573.5x, and the coverage for Hi-C and Chicago data is 1956.4x. More detailed information can be found in Supplementary data S1.

### *De novo* transcriptome assembly

The trimmed Hc1 Illumina RNA-seq data were assembled using Trinity v2.5.1 (Grabherr *et al.* 2011) with the parameter –SS_lib_type RF. Transcripts that contained adapters or vector contamination in the NCBI contamination database were removed. The assembly is available on the NCBI Transcriptome Shotgun Assembly archive, accession GHXS00000000.

### Mitochondrial genome and phylogeny

We assembled the mitochondrial genomes of *H. californensis* individuals Hc1 and Hc2 using PacBio and Illumina reads, using canu v2.1.1 (Koren *et al*. 2017) and pilon v1.22 (Walker *et al*. 2014). To determine the phylogenetic position of *H. californensis* individuals Hc1 and Hc2 we constructed an 18S tree, a COX1 nucleotide tree, and a multi-locus mitochondrial protein tree. See the Supplementary materials sections *Mitochondrial Genome Assembly and Annotation*, and *Phylogeny construction*.

### Genome assembly

#### Genome size estimation

K-mers were counted from trimmed Illumina WGS *H. californensis* reads and from publicly available *P. bachei* WGS libraries (SRR116669 and SRR116670) (Moroz *et al.* 2014) using jellyfish v2.2.10 (Marçais and Kingsford 2011) with options -C-s1000000000-k21. Genome sizes of both species were estimated using the *k*-mer count histograms on the GenomeScope2 server (Ranallo-Benavidez *et al.* 2020).

#### Genome assembly

The genome was assembled using wtdbg2 v2.4 (Ruan and Li 2019). The assembly was then polished with arrow v2.2 (github.com/PacificBiosciences/gcpp), then with pilon v1.22 (Walker *et al.* 2014). Haplotigs were removed with Purge Haplotigs v1.0.4 (Roach *et al.* 2018). Dovetail Genomics HiRise vAug2019 was used to scaffold the haplotig-purged assembly with the trimmed Chicago and Hi-C reads. Scaffolds with a mean coverage of less than 100, or having greater than 50% GC, were removed from the assembly using BlobTools v1.1.1 (Laetsch and Blaxter 2017). Assembly gaps were closed with LRGapcloser (Xu *et al*. 2019). The assembly was polished with pilon. See the *Genome Assembly* section of the Supplementary methods for additional details.

#### Genome quality assessment

We calculated the final assembly statistics such as the number of scaffolds, contigs, and the N50, using the program fasta_stats included with the Meraculous assembler (Chapman *et al.* 2011). We also assessed the completeness of the assembly by calculating the percent of PacBio Sequel subreads and full-length nonchimeric (FLNC) transcripts that mapped to the assembly. We also used a custom python script to calculate the percent of bases of each read type that mapped to the assembly. We performed a self-to-self genome alignment using LASTZ v1.04.03 (Harris 2007) to check for erroneously duplicated regions. To check for uncollapsed haplotypes or regions with many indels we used samtools mpileup v1.7 (Li *et al.* 2009) and chep commit 60c4312 (github.com/conchoecia/chep).

#### Characterizing chromosomal inversions

We generated a Hi-C heatmap to check for genome misassemblies. For details, see the *Hi-C heatmap generation* Supplementary section. We noticed three strong off-diagonal bowtie-shaped Hi-C hotspots on Chromosome 1. If this type of signal arises from a misassembly, then the misassembly can be corrected by inverting the bowtie-shaped region of the Hi-C matrix. Heterozygous inversions are not correctable by the same process (Corbett-Detig *et al.* 2019; Chida *et al.* 2020). We used PretextView to combinatorially invert sections of the Hi-C matrix to attempt to remove the off-diagonal signal.

#### Genome variant calling and phasing

To find diploid variants in the genome, we mapped PacBio CLR and Illumina WGS reads to the genome with minimap2 v2.17 (Li 2017) and BWA-MEM v0.7.17 (Li 2013), called variants using freebayes v1.3.2-38 (Garrison and Marth 2012) and gnu parallel v20161222 (Tange 2011), then filtered the VCF to only include diploid calls. We then phased the variants using Picard v2.25.1 (“Picard Toolkit” 2016) and HapCUT2 v1.3.1 (Edge *et al.* 2017). See the section *Genome Variant Calling and Phasing* in the Supplementary methods for parameters.

### Genome annotation

#### Manual genome annotation

We annotated the genome by manually curating transcript models generated from several datasets. The transcript sets were generated with PacBio Iso-Seq and Illumina RNA-seq reads as input for BRAKER v2.14 (Hoff *et al.* 2019), AUGUSTUS v3.3.3 (Stanke *et al.* 2004), and GeneMark-ES/ET v4.65 (Hoff *et al.* 2016). The PacBio Iso-Seq data were used as input for PacBio Cupcake tools v8.0 (github.com/Magdoll/cDNA_Cupcake), StringTie v2.0.4 (Pertea *et al.* 2015), and Pinfish commit b6f3c06 (github.com/nanoporetech/pinfish). See the *Genome Annotation and Transcript phasing* sections of the Supplementary materials for details on how each program was run.

Genome annotation consisted of three rounds. In annotation round 1, we reviewed the genome in IGV or JBrowse and manually verified the StringTie transcripts that were generated with the PacBio Iso-Seq data. Specifically, we identified if the StringTie transcripts were fused, correct, or fragmented by comparing them to the FLNC PacBio Iso-Seq reads. If the mapping pattern of PacBio Iso-seq reads suggested that a StringTie transcript was a fusion between two or more adjacent transcripts, then we replaced the fused StringTie transcript with Pinfish, AUGUSTUS, or GeneMark-ES/ET transcripts. The replacement transcripts were selected if they matched the gene structure of the PacBio Iso-Seq reads that mapped to the same locus. If a StringTie transcript was only a partial gene, also evidenced by the PacBio Iso-Seq reads, then the partial StringTie transcript was replaced with a correct Pinfish, AUGUSTUS, or GeneMark-ES/ET transcript. StringTie transcripts that did not match a transcript observed in the PacBio Iso-Seq data were removed. If Iso-Seq reads were mapped to a locus, but the locus had no representative StringTie transcript, then a matching Pinfish, AUGUSTUS, or GeneMark-ES/ET transcript was added to the annotation. StringTie transcripts that were grouped together by StringTie, but actually represented multiple genes with mutually exclusive exons, were split into multiple genes. At this stage the annotation contained genes and transcripts representing the complement of PacBio Iso-Seq data derived from the adult Hc1.

In annotation round 2, AUGUSTUS gene models generated from hints that included Illumina RNA-seq reads were added to the annotation if they did not overlap with the transcripts from round 1.

In annotation round 3, we sought to find life-stage-specific and tissue-specific transcripts in the *H. californensis* genome that may not have been present in the RNA sample from the adult Hc1. Gene models were generated by mapping *P. bachei* transcripts to the *H. californensis* genome. The resulting gene models were removed if they did not contain an ORF in the *H. californensis* genome, or if they overlapped with *H. californensis* annotation round 1 or round 2 genes. Gene models were only included in the annotation if their ORF’s protein product had a blastp v2.10.0+ (Altschul *et al*. 1997) hit to publicly available ctenophore transcriptomes with an *e*-value of less than 1e-10.

For each transcript in the annotation, we generated haplotype-resolved protein sequences. See the *Genome Annotation and Transcript phasing* sections of the Supplementary materials for more details.

#### Annotation completeness assessment

We used gVolante and BUSCO Eukaryota v3 to calculate the BUSCO score of the protein models from our annotation, the *de novo* transcriptome, and the genome assembly (Simão *et al.* 2015; Nishimura *et al.* 2017).

#### TAD calling and boundary analysis

We called topologically associating domains (TADs) using the HOMER Hi-C analysis pipeline (Heinz *et al.* 2018). The TADs were called with 1/4 kb bin resolutions and 10/40 kb windows. We masked regions around Hi-C heatmap irregularities such as off-diagonal signal that appeared to be due to inversions. This signal confounds the discovery of TADs in well-assembled genome regions. We calculated TAD separation scores with HiCExplorer v3.6 (Ramírez *et al.* 2018) using Cooler v0.8.10 (Abdennur and Mirny 2020).

We applied HOMER’s *de novo* motif discovery pipeline to 1.5 kb regions on either side of each TAD boundary (Heinz *et al.* 2018). For motif discovery, we selected background regions that exhibited minimal local change in TAD separation score, as these regions least resemble TAD boundaries.

We noticed that TADs tended to occur near gene boundaries. To test the significance of this observation we performed a permutation test. We first measured the median distance between TAD boundaries and the nearest gene. The background distribution was calculated by 1000 permutations of randomly placing TADs across the genome using the same size distribution as our observed TADs, then measuring the distance to the nearest gene.

#### Identification of nested intronic genes

Nested intronic genes were identified using chep gff_to_intron_bed.py (github.com/conchoecia/chep, commit 60c4312), allowing for a 15% overlap with the host exons at both the 5ʹ and 3ʹ ends of the nested transcript. We excluded the longest 0.5% of introns from the analysis to avoid counting the introns from trans-spliced splice leaders.

#### Repeats and centromeres

We used Tandem Repeats Finder v March 13, 2006 (Benson 1999) and EDTA v1.8.3 (Ou *et al.* 2019) to identify repeats and transposable elements.

### Comparative analyses

#### Whole-genome heterozygosity estimation

The single nucleotide heterozygosity of Hc1 was estimated by only using sites that had exactly 178x Illumina WGS read mapping depth. This depth, 178x, was the mode of the mapping depth for the whole genome, and thus represents sites at which reads from both haplotypes mapped. We implemented this method, first described in Saremi *et al.* (2019), in a purpose-built software package called chep (github.com/conchoecia/chep). We also measured the heterozygosity of the Hc1 exonic, intronic, and intergenic regions on individual chromosomes using chep.

We measured the heterozygosity of Hc1, Hc2, and *P. bachei* individual SAMN00216730 by counting 21-mers with jellyfish v2.2.10 (Marçais and Kingsford 2011) then using the resulting spectrum in GenomeScope 2 (Ranallo-Benavidez *et al.* 2020), by using vcftools’ –het option (v0.1.17), and by using angsd realSFS (v0.921) (Danecek *et al.* 2011; Korneliussen *et al.* 2014). We were not able to measure the heterozygosity of *M. leidyi* because the sequencing libraries were derived from multiple individuals.

#### Analysis of the HiC-scaffolded Pleurobrachia genome

The *P.* *bachei* genome assembly was recently scaffolded using Hi-C data (Hoencamp *et al.* 2021). The new, scaffolded assembly did not include a genome annotation. We identified the protein positions in the *P. bachei* genome assembly using tblastn and the previously published *P. bachei* protein sequences (Moroz *et al.* 2014). We also looked for orthologous scaffolds between the *H. californensis and P. bachei* genomes by plotting the protein coordinates of reciprocal best blastp hits between the proteins of the two genomes. For comparative analysis, we generated a *P. bachei* Hi-C heatmap by mapping the *P. bachei* Hi-C reads, SRR13364273 (Hoencamp *et al.* 2021), to the new assembly using the same protocol as for *H. californensis*. See the *Hi-C heatmap generation* Supplementary section for details.

#### Microsynteny between ctenophores

The *H. californensis* proteins were queried against the *M. leidyi and P. bachei* proteins using diamond blastp v0.9.24 (Buchfink *et al.* 2015). The gene positions and the diamond blastp table were then used to identify collinear blocks of genes using the purpose-built Python script microsynteny.py (github.com/wrf/genomeGTFtools). We required a minimum of 3 consecutive genes, allowed for up to 5 intervening genes, and allowed a maximum distance of 30 kb to the next gene.

## Results and discussion

### Genome sequencing and assembly

To determine the ploidy of *H. californensis* and to estimate its genome size, we computed *k*-mer spectra from *H. californensis and P. bachei* WGS libraries. Libraries from both species had two major *k*-mer peaks. The lower-coverage peak was larger than the higher-coverage peak in both species. This pattern is consistent with the *k*-mer spectra of other highly heterozygous diploid organisms (Ranallo-Benavidez *et al.* 2020). From the k-mer spectrum, the predicted 1C genome size of *H. californensis* was 96–98 Mb (Supplementary Figure S4), which is close to the predicted *P. bachei* genome size, 97.5 Mb (Supplementary Figure S5).

We aimed to generate a chromosome-scale reference genome for *H. californensis* in which each chromosome is represented by a composite sequence obtained by combining both haplotypes. This genome sequence was assembled from PacBio long reads, polished with Illumina short paired-end reads, and scaffolded with *in vitro and in vivo* chromatin conformation capture reads from a single individual, Hc1 (Methods). The *H. californensis* genome assembly totaled 110.6 Mb in 44 scaffolds and 351 contigs. Half of the sequence is present in scaffolds longer than 8.5 Mb (N50), with 2.76 gaps per Mb within scaffolds. The 13 longest scaffolds comprise 99.47% of the assembly, ranging in size from 10.3 to 6.4 Mb. These 13 long scaffolds match the microscopy-based karyotype of *n* = 13, detailed below. The remaining 31 scaffolds were each shorter than 50 kb and represent short unplaced sequences. We found no detectable contamination from marine bacteria or gut contents based on the blobtools results (Supplementary Figure S6). The genome dotplot made with D-Genies (Cabanettes and Klopp 2018) did not reveal erroneously duplicated assembly regions (Supplementary Figure S7). 95.32% of the PacBio Sequel subreads (Supplementary Table S1), and 99.02% of PacBio Sequel II Iso-Seq FLNC transcripts mapped to the 13 largest scaffolds.

We note that our 110 Mb *H. californensis* assembly is substantially shorter than the published *P. bachei* assembly (156 Mb) despite similar size estimates based on *k*-mer spectra. Our analysis of the *P. bachei* assembly, included in the Supplemental text, suggests that over half of the reported *P. bachei* scaffolds are unmerged haplotypes.

### Variant calling and phasing

We called variants using freebayes after mapping Hc1 PacBio CLR and Illumina WGS reads to the *H. californensis* reference genome. After filtering we identified 2.24 million heterozygous single nucleotide or indel variants. These variants were phased using PacBio CLR, Chicago, and Hi-C reads, resulting in phased blocks of variants that spanned more than 99% of the length of each chromosome-length scaffold. Of the 2.24 million diploid variants, 1.75 million (77.9%) were in the chromosome-scale phased variant blocks. The high density of phased variants, one for every 63 bp of genome assembly, suggests that the *H. californensis* data may be a useful benchmarking candidate for phased, or diploid, genome assemblers.

### Mitochondrial genome and phylogeny

We assembled and annotated the mitochondrial genomes of Hc1 and Hc2, two individuals from the same Monterey Bay population, and collected 3 years apart. The mitochondrial genomes (mtDNA) from Hc1 and Hc2 were 99.6% identical. The *H. californensis* mtDNA is 71.5% identical to the mtDNA of the closely related *P. bachei*, and 80% identical to *P. bachei* when only considering coding regions. The *H. californensis* mitochondrial genome has a 1.8 kb insertion relative to *P. bachei*, between COX2 and 16S (Supplementary Figure S8). The percent identity between the *H. californensis and P. bachei* mtDNA confirms they are distinct species, despite their similar morphology. Phylogenetic analysis of *P. bachei* and *H. californensis* mtDNA is also consistent with these being distinct but closely related species (Supplementary Figure S9).

Despite the distinct mtDNA of *H. californensis and P. bachei*, phylogenetic trees based on 18S rRNA and COX1 show that *H. californensis* falls within the *Pleurobrachia* clade. Furthermore, other *Hormiphora* species are sister to *Pleurobrachia*. These results suggest that the genus *Hormiphora* as currently defined may be polyphyletic. Future taxonomy work should consider reassigning *H. californensis* to the genus *Pleurobrachia*.

### Karyotype

**Figure 1:**
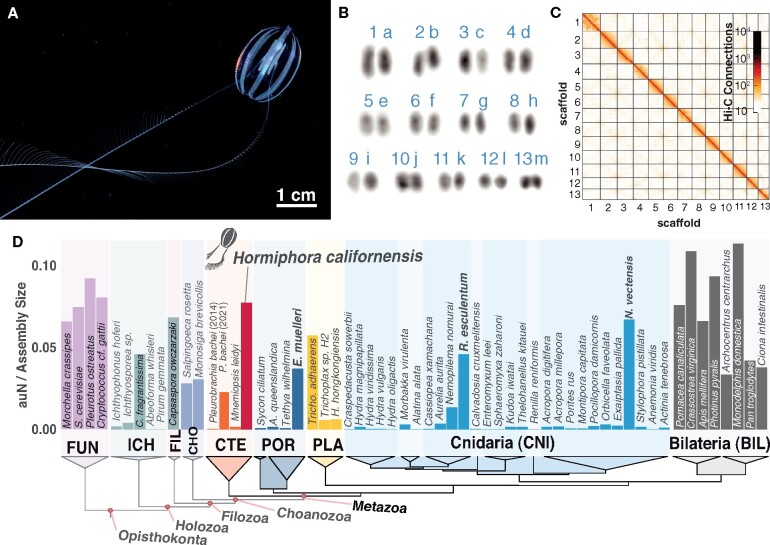
Karyotype and genome assembly quality. (A) *H. californensis* with its tentacles extended during feeding. (B) One karyotype image (rearranged and color-inverted) of a *H. californensis* chromosome preparation that contained 13 chromosome pairs. (C) The *H. californensis* Hi-C map, showing thirteen chromosome-scale scaffolds. (D) Genome contiguity normalized by genome size [auN/Assembly Size, per (Li 2020)] from all available nonbilaterian holozoan genomes and select fungal and bilaterian genomes, with tree topology based on (Whelan *et al.* 2017). Bolded species names are nonbilaterians with chromosome-scale genome assemblies. BIL, bilaterians; CHO, choanoflagellates; CNI, cnidarians; CTE, ctenophores; FIL, Filasteria; FUN, Fungi; ICH, Ichthyosporea; PLA, placozoans; POR, Porifera.

The karyotype has not been previously described for any ctenophore species. We used microscopy of DAPI-stained chromosome spreads to determine that the *H. californensis* genome is composed of *n* = 13 chromosome pairs (Figure 1B, Supplementary Figure S10). Four of the images correspond to a 2n of 26, and the remaining images have counts within 3 chromosomes of 26. This count, 13 pairs, is consistent with the 13 multi-megabase scaffolds in the *de novo* genome assembly presented here.

### Genome annotation

We manually annotated the genome using gene models generated with Hc1 Iso-Seq reads, Illumina RNA-seq data, and *P. bachei* transcripts. The long Iso-Seq reads capture, in many cases, complete cDNA sequences and represent transcripts from a single haplotype. These features allowed us to produce haplotype-specific transcripts and proteins.

Our approach to annotating the *H. californensis* genome identified 14,265 protein-coding genes, of which 13,235 are supported by Iso-Seq reads (Supplementary Table S2). The BUSCO complete plus fragmented score was 96% (303 Eukaryotic genes— Supplementary Table S3). We found that 96% of the *Pleurobrachia* proteins with orthologs in other ctenophores also have an ortholog in the *H. californensis* annotation. We note that due to the haplotype redundancy of the *P. bachei* assembly, many annotated *P. bachei* genes are reported in allelic copies, which therefore overestimates the gene count of this species (Supplementary materials).

In addition, the *H. californensis* genome contains 619 protein-coding genes that have orthologs in other ctenophore transcriptomes, but do not appear in either the *M. leidyi* or *P. bachei* genomes (Supplementary Table S2). Of those 619 genes, 122 had blastp hits to nr, and included genes with a wide variety of functions such as DNA-binding proteins, calmodulins, histones, proteases, and more. Among these 122 genes we did not find any evidence for the presence of the neural and mesoderm-component genes reported to be missing from ctenophores (Ryan *et al.* 2013).

We found 1729 cases where two or more neighboring *P. bachei* gene models, and 1200 cases where *M. leidyi* gene models, appear to be fragments of a larger gene based on orthology with *H. californensis*. For example, the pecanex gene (2096 amino acids in *H. californensis*) appears to be split into four proteins in the *M. leidyi* annotation (Supplementary Figure S11).

97.7% of the eukaryotic BUSCOs were complete or partial in the translated Iso-Seq FLNC data, and 99.0% were complete or partial in the Illumina RNA-seq *de novo* transcriptome. Because these values are higher than the 96% complete or partial BUSCOs from the genome annotation, it is possible that the genome annotation does not capture the full complement of *H. californensis* genes. Future annotation iterations will benefit from Iso-Seq sequencing of different tissues and developmental stages.

Tandem Repeats Finder (Benson 1999) identified 14 Mb (13%) of the genome as repeats, none of which were identifiable as centromeric. Thus, we are unable to annotate or further describe centromeres in these genomes.

### Topologically associating domains and 3D genome structure

Genome analyses using Hi-C data have shown that in many species, chromatin is organized in segments of close proximity that are known as TADs (Lieberman-Aiden *et al.* 2009).

We used proximity ligation data to identify and characterize TADs in *H. californensis* and found evidence that the *H. californensis* genome contains small TADs with a median length of 60 kb. The *H. californensis* TADs are significantly smaller than human TADs (median length 1.15 Mb) (McArthur and Capra 2021). Despite the fact that the *Ephydatia and Drosophila* genomes are comparable in size to the *H. californensis* genome, the mean *H. californensis* TAD length is half the length of the TADs in those two species (Hou *et al.* 2012; Kenny *et al.* 2020). We found that TAD boundaries tend to occur in the noncoding DNA bordering genes (*P* = 0.001, permutation). The mean distance from a TAD boundary to a gene is 7.8 kb.

We used HOMER to search, *de novo*, for DNA motifs in the sequences flanking the outside of TADs. In these sequences flanking TADs we found enriched motifs, most of which resembled the RNA polymerase II-binding motifs of known transcription factors. The six most-enriched motifs were homeodomain and *MYB*-related transcription factor binding sites, which are conserved in eukaryotes. Homeodomain and *MYB*-related transcription factor genes, as well as RNA polymerase II, were present in the *H. californensis* genome annotation.

### Analysis of the HiC-scaffolded Pleurobrachia genome

We assessed the quality of the *P. bachei* genome assembly that was recently scaffolded using Hi-C data (Hoencamp *et al.* 2021). This assembly was generated by linking together contigs and scaffolds from the original *P. bachei* assembly (Moroz *et al.* 2014). The authors reported that they found 13 or more putative chromosomal scaffolds, but did not provide further description or analysis of the assembly.

The scaffolded *P. bachei* genome contains 157.1 Mbp in 20,121 scaffolds and 39,072 contigs. The 13 largest scaffolds contain 81.5 Mb of sequence, and appear to be chromosome-scale in the Hi-C map. Those scaffolds contain 69.4 Mb of contigs, and 12.2 Mb of stretches of Ns. Given that the predicted 1C size of the *P. bachei* genome is 96.6 Mbp (see results above), the assembly size of the contigs in the 13 largest scaffolds relative to the predicted size is 72%. The 81.5 Mbp in the 13 largest scaffolds is 51.9% of the total assembly size (Supplementary Figure S12).

To further investigate the completeness and correctness of the 13 chromosome-scale *P. bachei* scaffolds, we performed synteny comparisons with the *H. californensis* genome assembly. The Moroz *et al.* (2014) and Hoencamp *et al.* (2021)*P. bachei* genomes do not include genome annotations, although Moroz *et al.* (2014) included 19,002 *P. bachei* protein models from the genome sequence. Using those 19,002 proteins we identified 9714 genes on the 13 largest *P. bachei* scaffolds. This is 68.2% of the total number of genes found on the 13 largest *Hormiphora* scaffolds. We performed a reciprocal-best blastp search between the *P. bachei and H. californensis* proteins and plotted their coordinates in 2-dimensions to visualize regions of macrosynteny, also called an Oxford dot plot. This plot revealed that each of the 13 largest *P. bachei and H. californensis* scaffolds predominantly had reciprocal best blastp hits on only one scaffold of the other species (Supplementary Figure S13). We did not find evidence for chromosomal fusions or fissions between the karyotype of the two animals.

In summary, we find that the scaffolded *P. bachei* assembly contains 13 scaffolds that correspond to the 13 chromosomes of *H. californensis*. However, as a large fraction of the *P. bachei* genome is not represented in these 13 scaffolds as measured by overall assembled genome sequence or gene content, the *P. bachei* genome would likely be greatly improved using a contemporary long-read contig assembly approach, followed by chromosome-scale scaffolding.

### Heterozygous chromosome inversions and microsynteny

**Figure 2:**
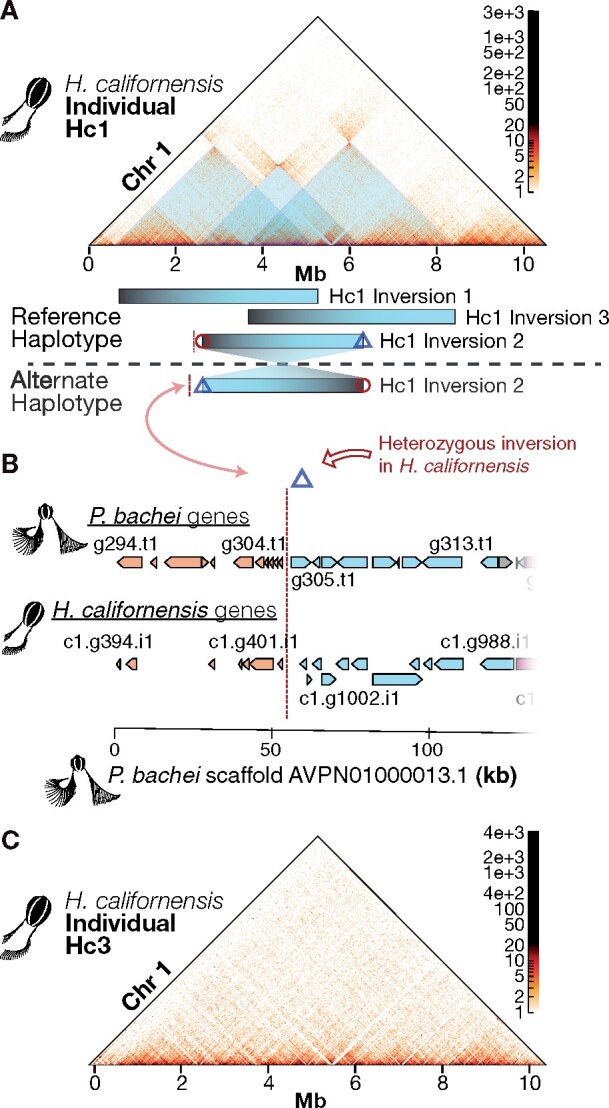
Heterozygous inversions on *H. californensis* chromosome 1. (A) Three heterozygous overlapping inversions are present on chromosome 1 in the Hc1 genome. Black/blue bars show the spans of each heterozygous inversion. (B) The alternative haplotype of Hc1 inversion 2, indicated by the close proximity of the blue diamond and red line, has the same gene order as the *P. bachei genome*. The *x*-axis is genome coordinates of *P. bachei* scaffold AVPN01000013.1. Orange *H. californensis and P. bachei* genes to the left of the vertical red dotted line are orthologous and have microsynteny. Blue genes to the right of the vertical red dotted line are orthologous and have microsynteny. (C) Hi-C map of *H. californensis* individual Hc3 shows concordance with Hc1 chromosome 1, but no off-diagonal Hi-C evidence for heterozygous inversions.

Chromosome 1 of *H. californensis* individual Hc1 contains three large heterozygous chromosomal inversions (Figure 2A, Supplementary Figure S14). Each inversion is approximately 2 Mbp, or 20% of chromosome 1. Together, these putative inversions span 73% of the length of chromosome 1. These are unlikely to be assembly errors, since inverting the Hi-C heatmap around the errors does not remove the off-diagonal signal (Supplementary Figure S14). All six breaks of between-haplotype synteny appear to occur between genes, and outside of TAD boundaries. The Hi-C matrix from individual Hc3 does not have off-diagonal hotspots (Figure 2C), suggesting that both haplotypes of Hc3 chromosome 1 match the genome assembly sequence. Large heterozygous inversions can prevent recombination over large chromosomal regions (Kirkpatrick 2010; McBroome *et al.* 2020), therefore these two haplotypes of *H. californensis* chromosome 1 may be segregating independently. Large heterozygous inversions between the haplotypes in one individual are not prevalent in vertebrate species, but have been observed before in the genomes of other invertebrates, such as in the mosquito *Anopheles gambiae* (Corbett-Detig *et al.* 2019).

We examined whether the inversion breakpoints in *H. californensis* chromosome 1 also occurred in the genome of the closely related *P. bachei.* Three of these breakpoints, including an exact match to Hc1 heterozygous inversion 2, were found to occur in the *P. bachei* genome. Hc1 inversion 2, in which *H. californensis* gene 355 on chromosome 1 lies next to *H. californensis* gene 864 (sequentially numbered) on the alternate haplotype, reflects the gene order in the *P. bachei* genome on scaffold AVPN01000013.1 (Figure 2B). The *H. californensis* inversion breakpoint at position 5.20 Mb is also a point of synteny mismatch in *P. bachei*, in which the gene on one side of the *P. bachei* synteny break matches a synteny breakpoint in *H. californensis* (*H. californensis* gene c1.g741). However, the gene on the opposite side of the synteny break (*H. californensis* gene c1.g424) does not match any of the gene intervals from inversions 1, 2, or 3 from *H. californensis*. These results suggest that chromosomal inversions may not only exist between different ctenophore species, but also may be prevalent within a single population of one species.

Gene colinearity analyses suggest that *H. californensis and M. leidyi* only share limited gene microsynteny. The largest identifiable blocks of gene colinearity only contained four genes in common. Given the extensive gene rearrangements seen between the closely related species *H. californensis and P. bachei*, it is not surprising to find the lack of gene colinearity between the distantly related *H. californensis and M. leidyi*.

The largest colinear block was over 5.8 Mbp of *H.californensis*-*P.bachei* chromosome 5, encompassing 964 genes in *H. californensis*. However, most other chromosomes were significantly rearranged between the two species.

### Comparative analyses

#### Heterozygosity

**Figure 3:**
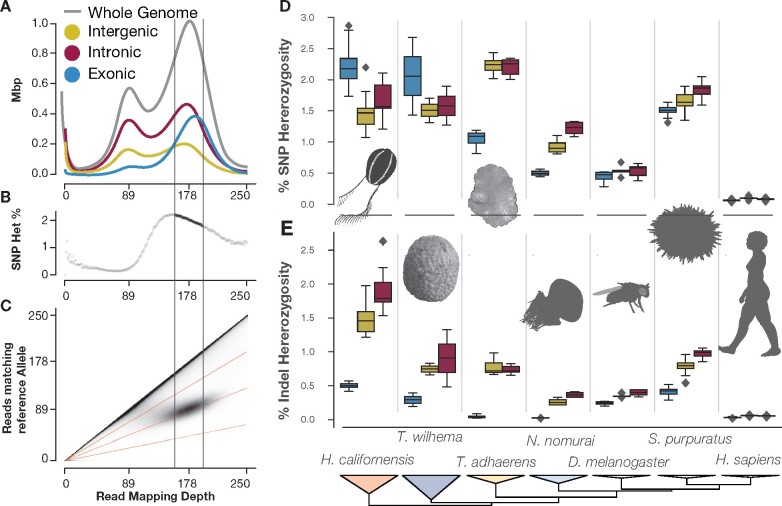
The *Hormiphora* genome is highly heterozygous and contains many indels in noncoding regions. (A) The histogram of the number of bases in a genome assembly (*y*-axis) with a specific mapping depth (*x*-axis) using Illumina WGS reads. The mode of the mapping depth of the Illumina WGS reads was 178x, so bases with close to 178x mapping coverage have reads from both haplotypes. Therefore, bases with a mapping coverage around 89x have reads mapped from a single haplotype. (B) The whole-genome heterozygosity calculated at each read mapping depth. The heterozygosity value for each mapping depth (*x*-axis) is the number of sites where only one half of the mapped reads match the reference allele, divided by the total number of sites at that depth. (C) The 2D histogram showing that bi-allelic sites are centered around 178x read mapping depth. Also, the 2D histogram is one way to visualize the data input for panel (B). (D–E) Box-and-whiskers plots show the distribution of SNP (D) or indel (E) heterozygosity in each chromosome-size scaffold. In *H. californensis*, the intergenic and intronic regions have a reduced SNP heterozygosity, but an elevated rate of indels. This pattern differs from the correlation between SNP and indel heterozygosity found in species with lower overall heterozygosity.

We measured the heterozygosity of the intronic, exonic, and intergenic regions of *H. californensis* and six other metazoan species (Figure 3, Supplementary Figure S15 and Tables S4 and S5) using a method that avoids mis-estimation due to genome assembly errors or inaccurate heterozygous site calls in a VCF file (Saremi *et al.* 2019). *H. californensis* had a high combined single nucleotide and indel heterozygosity rate—approximately 3.2% overall, and a per-chromosome rate of between 2.4% and 4.7%. The overall single-nucleotide heterozygosity was 2%. These analyses also revealed that both *H. californensis* and the sponge *Tethya wilhelma* (Mills *et al*. 2018) had high SNP heterozygosity in exons, but depressed SNP heterozygosity in both intergenic and intronic regions (Figure 3, D and E). This pattern is contrary to other species, where heterozygosity of the introns and intergenic regions is higher than in the exons. This pattern in our data is likely due to short Illumina reads from one allele not mapping to regions with high combined SNP and indel heterozygosity (Figure 3A), therefore placing an artificial ceiling on the measurable heterozygosity. Using long, accurate reads such as PacBio HiFi data, or measuring heterozygosity with a diploid genome assembly, should overcome these shortcomings.

#### Ubiquity of trans-spliced leader sequences

A 2010 study of ctenophore and cnidarian ESTs showed that these phyla have extensive 5′-capping trans-splicing (Derelle *et al.* 2010). However, this study lacked genomes to examine the origins of the leader sequence (Derelle *et al.* 2010). One prevalent feature of *H. californensis* genome organization was gene clusters sharing a 5′ exon, but otherwise having different exons, and seemingly nonoverlapping transcripts. The shared first exons were between 35-48bp long, and were all >90% identical to 5ʹGAGTTTCAAACTTTTCAACACTACTTTAAACAAATTAATTTGAG 3ʹ. We identified 718 of these leader sequences in the *H. californensis* genome. The leader sequence was found on 56% of our Iso-Seq reads. This appears to be the result of trans-splicing of a leader sequence (Boroni *et al.* 2018). The Iso-Seq reads lacking the leader sequence may be sheared at the 5′ end, as is common in full-length cDNA library preparation. Thus, 56% represents a lower bound for the true percentage of *H. californensis* mRNAs with trans-spliced leaders. The shared exons we identified in the genome may be a result of the leader sequences on the Iso-Seq reads being artifactually mapped to the nearest spliced-leader locus 5ʹ of the transcript in the genome assembly.

In *M.* *leidyi*, although it was not reported previously, we found several examples of gene clusters with shared first exons using a *de novo M. leidyi* transcriptome (SRX993241) mapped to the *M. leidyi* genome. The leader motif from Derelle *et al.* (2010) was also identified in the *M. leidyi* genome 491 times. Both the *M. leidyi* and *H. californensis* leader sequences end in a TGAG motif, part of a mostly conserved 5ʹAATTTGAG 3ʹ motif. Over half of the annotated transcripts in *H. californensis* begin with AG.

#### Nested intronic genes in the Metazoa

**Figure 4:**
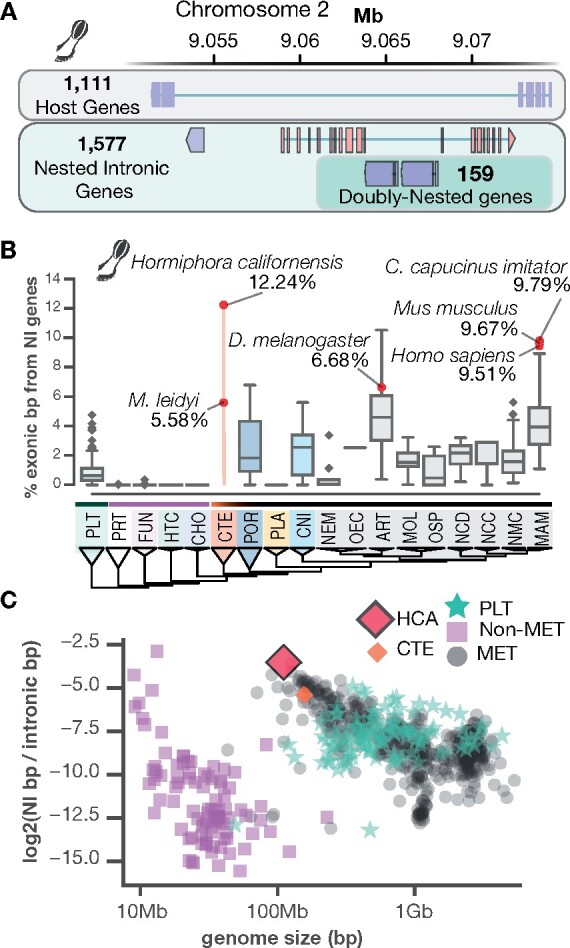
An analysis of NI genes in 1058 eukaryote genomes. (A) NI genes are genes that are contained within the introns of other genes. In this example from the *H. californensis* genome, four genes are nested within a single host gene. Two of the genes are doubly nested—they are within the intron of another NI gene. Blue genes are coded on the antisense strand relative to the reference sequence and red genes are coded on the sense strand. (B) The percent of all exonic bp that are in NI genes. Protists and nonmetazoans have a negligible proportion compared to animals. Abbreviations: ART, panarthropoda; CHO, choanoflagellates; CNI, cnidarians; CTE, ctenophores; FUN, fungi; HCA, *H. californensis*; HTC, holozoa through choanoflagellates; MAM, mammals; MET, Metazoa; MOL, molluscs; NCD, nonchordate deuterostomes; NCC, noncraniata chordates; NEM, nematodes; NMC, nonmammalian chordates; OEC, other ecdysozoa; OSP, other spiralians; PLA, placozoans; PLT, plants; POR, Porifera; PRT, other protists. (C) As animal and protist genomes become smaller, a higher proportion of the exonic bases are in NI genes.

Using 1058 eukaryotic genomes, including all genomes available on NCBI RefSeq, we quantified the percent of exonic basepairs that are from NI genes—genes whose transcripts are within the boundaries of a single intron of another gene (Figure 4). In the *H. californensis* genome, we found 1654 genes hosting one or more NI genes. There were 2357 NI genes inside the 1654 host genes (Supplementary data). We estimated that *H. californensis* has 12.24% of exonic bases in NI genes, similar to the rate found in primate and some arthropod genomes. From the 2357 NI genes we identified 484 doubly-nested genes, which are NI genes within another NI gene. We found that 1109 NI genes are flanked by transposable elements (TEs) on at least one side of the gene, and 176 NI genes are flanked on both sides by TEs. Many NI genes are also parallel with the host gene, necessitating a complex transcription or splicing system to separately process the two genes. Parallel NI genes have been observed before in other taxa, such as human (Yu *et al.* 2005) and fly (Henikoff *et al.* 1986).

We observed that NI genes are largely absent from the genomes of protists, fungi, and other nonmetazoan opisthokonts such as the choanoflagellates (Figure 4C). This observation could be due to genome annotation errors in those clades, or NI genes may have undergone genomic expansions in the metazoan last common ancestor (LCA), and in the plant LCA. We also found that smaller animal genomes tend to have a higher percent of exonic bases in NI genes (Figure 4C). Given that NI genes introduce additional ways to control transcription, such as antisense transcription competition (Yu *et al.* 2005), the punctuated appearance of these genes in the metazoa is possibly one of the complex transcriptional control mechanisms that evolved in the ancestor to all animals. High-quality genome assemblies and annotations of outgroup species to the metazoa will be necessary to determine the extent to which nested genes are a feature of metazoan genomes.

## Data availability

All sequencing reads, transcriptomes, and the genome assembly are available for download via NCBI BioProject PRJNA576068. Hc1 and Hc2 mitochondrial sequences are available through NCBI accessions MN544300 and MN544301. Additional data are available through G3 figshare: https://doi.org/10.25387/g3.15170382. Custom programs used in this publication, and annotation guidelines, are available at github.com/conchoecia/hormiphora and Zenodo DOI: 10.5281/zenodo.4074309. No tissue from these samples is available, as it was consumed during library preparation.

## Author Contributions

D.T.S., W.R.F., R.E.G., and S.H.D.H. conceived and designed the study. D.T.S. and L.M.C. collected sequencing data. D.T.S assembled the genome and transcriptomes. D.T.S and W.R.F. annotated the genome. D.T.S., W.R.F., and J.D.M. performed analyses on the data. D.T.S, W.R.F., J.D.M., and R.E.G. wrote the manuscript. All authors contributed to the revision and review of the manuscript.

## Funding

This work was supported by the David and Lucile Packard Foundation, the Monterey Bay Aquarium Research Institute, the University of California Biomolecular Engineering and Bioinformatics department; United States National Science Foundation DEB-1542679 to S.H.D.H.; the United States National Science Foundation GRFP DGE 1339067 to D.T.S.

## Conflicts of interest

R.E.G. is a cofounder and paid consultant of Dovetail Genomics. All other authors declare no competing interests.

## References

[jkab302-B1] Abdennur N , Mirny LA. 2020. Cooler: scalable storage for Hi-C data and other genomically labeled arrays. Bioinformatics. 36:311–316. 31290943 10.1093/bioinformatics/btz540 PMC8205516

[jkab302-B2] Adams M , McBroome J, Maurer N, Pepper-Tunick E, Saremi NF, et al 2020. One fly-one genome: chromosome-scale genome assembly of a single outbred *Drosophila melanogaster*. Nucleic Acids Res. 48:e75. 32491177 10.1093/nar/gkaa450 PMC7367183

[jkab302-B3] Altschul SF , Madden TL, Schäffer AA, Zhang J, Zhang Z, et al 1997. Gapped BLAST and PSI-BLAST: a new generation of protein database search programs. Nucleic Acids Res. 25:3389–3402. 9254694 10.1093/nar/25.17.3389 PMC146917

[jkab302-B103] Benson G . 1999. Tandem repeats finder: a program to analyze DNA sequences. Nucleic Acids Research. 27:2. 10.1093/nar/27.2.573 PMC148217 9862982

[jkab302-B6] Boroni M , Sammeth M, Gava SG, Jorge NAN, Macedo AM, et al 2018. Landscape of the spliced leader trans-splicing mechanism in *Schistosoma mansoni*. Sci Rep. 8:3877. 29497070 10.1038/s41598-018-22093-3 PMC5832876

[jkab302-B7] Bråte J , Neumann RS, Fromm B, Haraldsen AAB, Tarver JE, et al 2018. Unicellular origin of the animal MicroRNA machinery. Curr Biol. 28:3288–3295.e5. 30318349 10.1016/j.cub.2018.08.018 PMC6206976

[jkab302-B8] Buchfink B , Xie C, Huson DH. 2015. Fast and sensitive protein alignment using DIAMOND. Nat Methods. 12:59–60. 25402007 10.1038/nmeth.3176

[jkab302-B9] Burton JN , Adey A, Patwardhan RP, Qiu R, Kitzman JO, et al 2013. Chromosome-scale scaffolding of *de novo* genome assemblies based on chromatin interactions. Nat Biotechnol. 31:1119–1125. 24185095 10.1038/nbt.2727 PMC4117202

[jkab302-B10] Cabanettes F , Klopp C. 2018. D-GENIES: dot plot large genomes in an interactive, efficient and simple way. PeerJ. 6:e4958. 29888139 10.7717/peerj.4958 PMC5991294

[jkab302-B11] Chapman JA , Ho I, Sunkara S, Luo S, Schroth GP, et al 2011. Meraculous: *de novo* genome assembly with short paired-end reads. PLoS One. 6:e23501. 21876754 10.1371/journal.pone.0023501 PMC3158087

[jkab302-B12] Chida AR , Ravi S, Jayaprasad S, Paul K, Saha J, et al 2020. A near-chromosome level genome assembly of *Anopheles stephensi*. Front Genet. 11:565626. 33312190 10.3389/fgene.2020.565626 PMC7703621

[jkab302-B13] Corbett-Detig RB , Said I, Calzetta M, Genetti M, McBroome J, et al 2019. Fine-mapping complex inversion breakpoints and investigating somatic pairing in the *Anopheles gambiae* species complex using proximity-ligation sequencing. Genetics. 213:1495–1511. 31666292 10.1534/genetics.119.302385 PMC6893396

[jkab302-B14] Danecek P , Auton A, Abecasis G, Albers CA, Banks E, et al; 1000 Genomes Project Analysis Group. 2011. The variant call format and VCFtools. Bioinformatics. 27:2156–2158. 21653522 10.1093/bioinformatics/btr330 PMC3137218

[jkab302-B15] Dawson MN , Raskoff KA, Jacobs DK. 1998. Field preservation of marine invertebrate tissue for DNA analyses. Mol Mar Biol Biotechnol. 7:145–152. 11541322

[jkab302-B17] Derelle R , Momose T, Manuel M, Da Silva C, Wincker P, et al 2010. Convergent origins and rapid evolution of spliced leader trans-splicing in Metazoa: insights from the Ctenophora and Hydrozoa. RNA. 16:696–707. 20142326 10.1261/rna.1975210 PMC2844618

[jkab302-B19] Edge P , Bafna V, Bansal V. 2017. HapCUT2: robust and accurate haplotype assembly for diverse sequencing technologies. Genome Res. 27:801–812. 27940952 10.1101/gr.213462.116 PMC5411775

[jkab302-B20] Fernández R , Gabaldón T. 2020. Gene gain and loss across the metazoan tree of life. Nat Ecol Evol. 4:524–533. 31988444 10.1038/s41559-019-1069-x PMC7124887

[jkab302-B22] Freeman G. 1977. The establishment of the oral-aboral axis in the ctenophore embryo. Development. 42:237–260.

[jkab302-B23] Gaiti F , Calcino AD, Tanurdžić M, Degnan BM. 2017. Origin and evolution of the metazoan non-coding regulatory genome. Dev Biol. 427:193–202. 27880868 10.1016/j.ydbio.2016.11.013

[jkab302-B24] Garrison E , Marth G. 2012. Haplotype-based variant detection from short-read sequencing. arXiv. [q-bio.GN].

[jkab302-B25] Grabherr MG , Haas BJ, Yassour M, Levin JZ, Thompson DA, et al 2011. Full-length transcriptome assembly from RNA-Seq data without a reference genome. Nat Biotechnol. 29:644–652. 21572440 10.1038/nbt.1883 PMC3571712

[jkab302-B26] Guo L , Accorsi A, He S, Guerrero-Hernández C, Sivagnanam S, et al 2018. An adaptable chromosome preparation methodology for use in invertebrate research organisms. BMC Biol. 16:25. 29482548 10.1186/s12915-018-0497-4 PMC5828064

[jkab302-B27] Harris RS. 2007. Improved Pairwise Alignment of Genomic DNA. The Pennsylvania State University.

[jkab302-B28] Heinz S , Texari L, Hayes MGB, Urbanowski M, Chang MW, et al 2018. Transcription elongation can affect genome 3D Structure. Cell. 174:1522–1536.e22. 30146161 10.1016/j.cell.2018.07.047 PMC6130916

[jkab302-B29] Henikoff S , Keene MA, Fechtel K, Fristrom JW. 1986. Gene within a gene: nested Drosophila genes encode unrelated proteins on opposite DNA strands. Cell. 44:33–42. 3079672 10.1016/0092-8674(86)90482-4

[jkab302-B30] Hoencamp C , Dudchenko O, Elbatsh AMO, Brahmachari S, Raaijmakers JA, et al 2021. 3D genomics across the tree of life reveals condensin II as a determinant of architecture type. Science. 372:984–989. 34045355 10.1126/science.abe2218 PMC8172041

[jkab302-B31] Hoff KJ , Lange S, Lomsadze A, Borodovsky M, Stanke M. 2016. BRAKER1: Unsupervised RNA-Seq-Based Genome Annotation with GeneMark-ET and AUGUSTUS. Bioinformatics. 32:767–769. 26559507 10.1093/bioinformatics/btv661 PMC6078167

[jkab302-B32] Hoff KJ , Lomsadze A, Borodovsky M, Stanke M. 2019. Whole-genome annotation with BRAKER. Methods Mol Biol. 1962:65–95. 31020555 10.1007/978-1-4939-9173-0_5 PMC6635606

[jkab302-B33] Hou C , Li L, Qin ZS, Corces VG. 2012. Gene density, transcription, and insulators contribute to the partition of the *Drosophila* genome into physical domains. Mol Cell. 48:471–484. 23041285 10.1016/j.molcel.2012.08.031 PMC3496039

[jkab302-B34] Kajitani R , Toshimoto K, Noguchi H, Toyoda A, Ogura Y, et al 2014. Efficient *de novo* assembly of highly heterozygous genomes from whole-genome shotgun short reads. Genome Res. 24:1384–1395. 24755901 10.1101/gr.170720.113 PMC4120091

[jkab302-B36] Kenny NJ , Francis WR, Rivera-Vicéns RE, Juravel K, de Mendoza A, et al 2020. Tracing animal genomic evolution with the chromosomal-level assembly of the freshwater sponge *Ephydatia muelleri*. Nat Commun. 11:3676. 32719321 10.1038/s41467-020-17397-w PMC7385117

[jkab302-B38] Kirkpatrick M. 2010. How and why chromosome inversions evolve? PLoS Biol. 8:e1000501. 20927412 10.1371/journal.pbio.1000501 PMC2946949

[jkab302-B40] Koren S , Walenz BP, Berlin K, Miller JR, Bergman NH, et al 2017. Canu: scalable and accurate long-read assembly via adaptive k-mer weighting and repeat separation. Genome Res. 27:722–736. 28298431 10.1101/gr.215087.116 PMC5411767

[jkab302-B41] Korneliussen TS , Albrechtsen A, Nielsen R. 2014. ANGSD: analysis of next generation sequencing data. BMC Bioinformatics. 15:356. 25420514 10.1186/s12859-014-0356-4 PMC4248462

[jkab302-B43] Laetsch DR , Blaxter ML. 2017. BlobTools: Interrogation of genome assemblies. F1000Res. 6:1287.

[jkab302-B45] Laumer CE , Fernández R, Lemer S, Combosch D, Kocot KM, et al 2019. Revisiting metazoan phylogeny with genomic sampling of all phyla. Proc Biol Sci. 286:20190831. 31288696 10.1098/rspb.2019.0831 PMC6650721

[jkab302-B46] Leffler EM , Bullaughey K, Matute DR, Meyer WK, Ségurel L, et al 2012. Revisiting an old riddle: what determines genetic diversity levels within species? PLoS Biol. 10:e1001388. 22984349 10.1371/journal.pbio.1001388 PMC3439417

[jkab302-B48] Li H. 2013. Aligning sequence reads, clone sequences and assembly contigs with BWA-MEM. arXiv.

[jkab302-B49] Li H. 2017. Minimap2: pairwise alignment for nucleotide sequences. arXiv. 10.1093/bioinformatics/bty191 PMC6137996 29750242

[jkab302-B50] Li H. 2020. auN: a new metric to measure assembly contiguity. https://lh3.github.io/2020/04/08/a-new-metric-on-assembly-contiguity

[jkab302-B51] Li H , Handsaker B, Wysoker A, Fennell T, Ruan J, et al; 1000 Genome Project Data Processing Subgroup. 2009. The Sequence Alignment/Map format and SAMtools. Bioinformatics. 25:2078–2079. 19505943 10.1093/bioinformatics/btp352 PMC2723002

[jkab302-B52] Li Y , Gao L, Pan Y, Tian M, Li Y, et al 2020. Chromosome-level reference genome of the jellyfish *Rhopilema esculentum*. Gigascience. 9:giaa036. 32315029 10.1093/gigascience/giaa036 PMC7172023

[jkab302-B53] Lieberman-Aiden E , van Berkum NL, Williams L, Imakaev M, Ragoczy T, et al 2009. Comprehensive mapping of long-range interactions reveals folding principles of the human genome. Science. 326:289–293. 19815776 10.1126/science.1181369 PMC2858594

[jkab302-B56] Marçais G , Kingsford C. 2011. A fast, lock-free approach for efficient parallel counting of occurrences of k-mers. Bioinformatics. 27:764–770. 21217122 10.1093/bioinformatics/btr011 PMC3051319

[jkab302-B57] Matthews BJ , Vosshall LB. 2020. How to turn an organism into a model organism in 10 “easy” steps. J Exp Biol. 223:jeb218198. 32034051 10.1242/jeb.218198 PMC7790198

[jkab302-B58] McArthur E , Capra JA. 2021. Topologically associating domain boundaries that are stable across diverse cell types are evolutionarily constrained and enriched for heritability. Am J Hum Genet. 108:269–283. 33545030 10.1016/j.ajhg.2021.01.001 PMC7895846

[jkab302-B59] McBroome J , Liang D, Corbett-Detig R. 2020. Fine-scale position effects shape the distribution of inversion breakpoints in *Drosophila melanogaster*. Genome Biol Evol. 12:1378–1391. 32437518 10.1093/gbe/evaa103 PMC7487137

[jkab302-B60] Mills DB , Francis WR, Vargas S, Larsen M, Elemans CP, et al 2018. The last common ancestor of animals lacked the HIF pathway and respired in low-oxygen environments. eLife. 7:e31176. 29402379 10.7554/eLife.31176 PMC5800844

[jkab302-B61] Moroz LL , Kocot KM, Citarella MR, Dosung S, Norekian TP, et al 2014. The ctenophore genome and the evolutionary origins of neural systems. Nature. 510:109–114. 24847885 10.1038/nature13400 PMC4337882

[jkab302-B62] Nishimura O , Hara Y, Kuraku S. 2017. gVolante for standardizing completeness assessment of genome and transcriptome assemblies. Bioinformatics. 33:3635–3637. 29036533 10.1093/bioinformatics/btx445 PMC5870689

[jkab302-B63] Nong W , Cao J, Li Y, Qu Z, Sun J, et al 2020. Jellyfish genomes reveal distinct homeobox gene clusters and conservation of small RNA processing. Nat Commun. 11:3051. 32561724 10.1038/s41467-020-16801-9 PMC7305137

[jkab302-B64] Ou S , Su W, Liao Y, Chougule K, Agda JRA, et al 2019. Benchmarking transposable element annotation methods for creation of a streamlined, comprehensive pipeline. Genome Biol. 20:275. 31843001 10.1186/s13059-019-1905-y PMC6913007

[jkab302-B65] Patry WL , Bubel MK, Hansen C, Knowles T. 2019. Diffusion tubes: a method for the mass culture of ctenophores and other pelagic marine invertebrates. bioRxiv. 751099. 10.7717/peerj.8938 PMC7147435 32292660

[jkab302-B67] Pertea M , Pertea GM, Antonescu CM, Chang T-C, Mendell JT, et al 2015. StringTie enables improved reconstruction of a transcriptome from RNA-seq reads. Nat Biotechnol. 33:290–295. 25690850 10.1038/nbt.3122 PMC4643835

[jkab302-B69] Toolkit Picard , 2016. Broad institute, GitHub repository. https://github.com/broadinstitute/picard

[jkab302-B70] Presnell JS , Browne WE. 2021. Krüppel-like factor gene function in the ctenophore Mnemiopsis leidyi assessed by CRISPR/Cas9-mediated genome editing. Development. dev.199771. 10.1242/dev.199771 34373891

[jkab302-B71] Putnam NH , O'Connell BL, Stites JC, Rice BJ, Blanchette M, et al 2016. Chromosome-scale shotgun assembly using an *in vitro* method for long-range linkage. Genome Res. 26:342–350. 26848124 10.1101/gr.193474.115 PMC4772016

[jkab302-B72] Ramírez F , Bhardwaj V, Arrigoni L, Lam KC, Grüning BA, et al 2018. High-resolution TADs reveal DNA sequences underlying genome organization in flies. Nat Commun. 9:189. 29335486 10.1038/s41467-017-02525-w PMC5768762

[jkab302-B73] Ranallo-Benavidez TR , Jaron KS, Schatz MC. 2020. GenomeScope 2.0 and Smudgeplot for reference-free profiling of polyploid genomes. Nat Commun. 11:1432. 32188846 10.1038/s41467-020-14998-3 PMC7080791

[jkab302-B74] Rice ES , Green RE. 2019. New approaches for genome assembly and scaffolding. Annu Rev Anim Biosci. 7:17–40. 30485757 10.1146/annurev-animal-020518-115344

[jkab302-B76] Roach MJ , Schmidt SA, Borneman AR. 2018. Purge Haplotigs: allelic contig reassignment for third-gen diploid genome assemblies. BMC Bioinformatics. 19:460. 30497373 10.1186/s12859-018-2485-7 PMC6267036

[jkab302-B79] Ruan J , Li H. 2019. Fast and accurate long-read assembly with wtdbg2. bioRxiv. 530972. 10.1038/s41592-019-0669-3 PMC7004874 31819265

[jkab302-B80] Ryan JF , Pang K, Schnitzler CE, Nguyen A-D, Moreland RT, et al; NISC Comparative Sequencing Program. 2013. The genome of the ctenophore *Mnemiopsis leidyi* and its implications for cell type evolution. Science. 342:1242592. 24337300 10.1126/science.1242592 PMC3920664

[jkab302-B81] Sacerdot C , Louis A, Bon C, Berthelot C, Crollius HR. 2018. Chromosome evolution at the origin of the ancestral vertebrate genome. Genome Biol. 19:166. 30333059 10.1186/s13059-018-1559-1 PMC6193309

[jkab302-B83] Saremi NF , Supple MA, Byrne A, Cahill JA, Coutinho LL, et al 2019. Puma genomes from North and South America provide insights into the genomic consequences of inbreeding. Nat Commun. 10:4769. 31628318 10.1038/s41467-019-12741-1 PMC6800433

[jkab302-B84] Sebé-Pedrós A , Chomsky E, Pang K, Lara-Astiaso D, Gaiti F, et al 2018. Early metazoan cell type diversity and the evolution of multicellular gene regulation. Nat Ecol Evol. 2:1176–1188. 29942020 10.1038/s41559-018-0575-6 PMC6040636

[jkab302-B85] Shen X-X , Hittinger CT, Rokas A, Minh BQ, Braun EL. 2017. Contentious relationships in phylogenomic studies can be driven by a handful of genes. Nat Ecol Evol. 1:126. 28812701 10.1038/s41559-017-0126 PMC5560076

[jkab302-B86] Simão FA , Waterhouse RM, Ioannidis P, Kriventseva EV, Zdobnov EM. 2015. BUSCO: assessing genome assembly and annotation completeness with single-copy orthologs. Bioinformatics. 31:3210–3212. 26059717 10.1093/bioinformatics/btv351

[jkab302-B87] Simion P , Philippe H, Baurain D, Jager M, Richter DJ, et al 2017. A large and consistent phylogenomic dataset supports sponges as the sister group to all other animals. Curr Biol. 27:958–967. 28318975 10.1016/j.cub.2017.02.031

[jkab302-B88] Simister RL , Schmitt S, Taylor MW. 2011. Evaluating methods for the preservation and extraction of DNA and RNA for analysis of microbial communities in marine sponges. J Exp Mar Bio Ecol. 397:38–43.

[jkab302-B91] Stanke M , Steinkamp R, Waack S, Morgenstern B. 2004. AUGUSTUS: a web server for gene finding in eukaryotes. Nucleic Acids Res. 32:W309–W312. 15215400 10.1093/nar/gkh379 PMC441517

[jkab302-B92] Tange O. 2011. Gnu parallel-the command-line power tool. USENIX Magazine. 36:42–47.

[jkab302-B93] Tikhonenkov DV , Hehenberger E, Esaulov AS, Belyakova OI, Mazei YA, et al 2020. Insights into the origin of metazoan multicellularity from predatory unicellular relatives of animals. BMC Biol. 18:39. 32272915 10.1186/s12915-020-0762-1 PMC7147346

[jkab302-B94] Walker BJ , Abeel T, Shea T, Priest M, Abouelliel A, et al 2014. Pilon: an integrated tool for comprehensive microbial variant detection and genome assembly improvement. PLoS One. 9:e112963. 25409509 10.1371/journal.pone.0112963 PMC4237348

[jkab302-B95] Wang J , Zhang L, Lian S, Qin Z, Zhu X, et al 2020. Evolutionary transcriptomics of metazoan biphasic life cycle supports a single intercalation origin of metazoan larvae. Nat Ecol Evol. 4:725–736. 32203475 10.1038/s41559-020-1138-1

[jkab302-B98] Whelan NV , Kocot KM, Moroz TP, Mukherjee K, Williams P, et al 2017. Ctenophore relationships and their placement as the sister group to all other animals. Nat Ecol Evol. 1:1737–1746. 28993654 10.1038/s41559-017-0331-3 PMC5664179

[jkab302-B99] Xu G-C , Xu T-J, Zhu R, Zhang Y, Li S-Q, et al 2019. LR_Gapcloser: a tiling path-based gap closer that uses long reads to complete genome assembly. Gigascience. 8: 1. 10.1093/gigascience/giy157 PMC6324547 30576505

[jkab302-B101] Yu P , Ma D, Xu M. 2005. Nested genes in the human genome. Genomics. 86:414–422. 16084061 10.1016/j.ygeno.2005.06.008

[jkab302-B102] Zimmermann B , Robb SMC, Genikhovich G, Fropf WJ, Weilguny L, et al 2020. Sea anemone genomes reveal ancestral metazoan chromosomal macrosynteny. bioRxiv. 10.30.359448.

